# Supplementary material for: Mechanical sensing protein PIEZO1 regulates bone homeostasis via osteoblast-osteoclast crosstalk
Source: Nat Commun. 2020 Jan 15;11:282. doi: 10.1038/s41467-019-14146-6 (PMC6962448; doi:10.1038/s41467-019-14146-6)
Supplement: Supplementary file 1 — Supplementary Information [file 41467_2019_14146_MOESM1_ESM.pdf]

Supplementary Information

Mechanical sensing protein PIEZO1 regulates bone homeostasis via osteoblast-osteoclast crosstalk

Wang et al.

Supplementary Information contains

Supplementary Figures 1 – 11

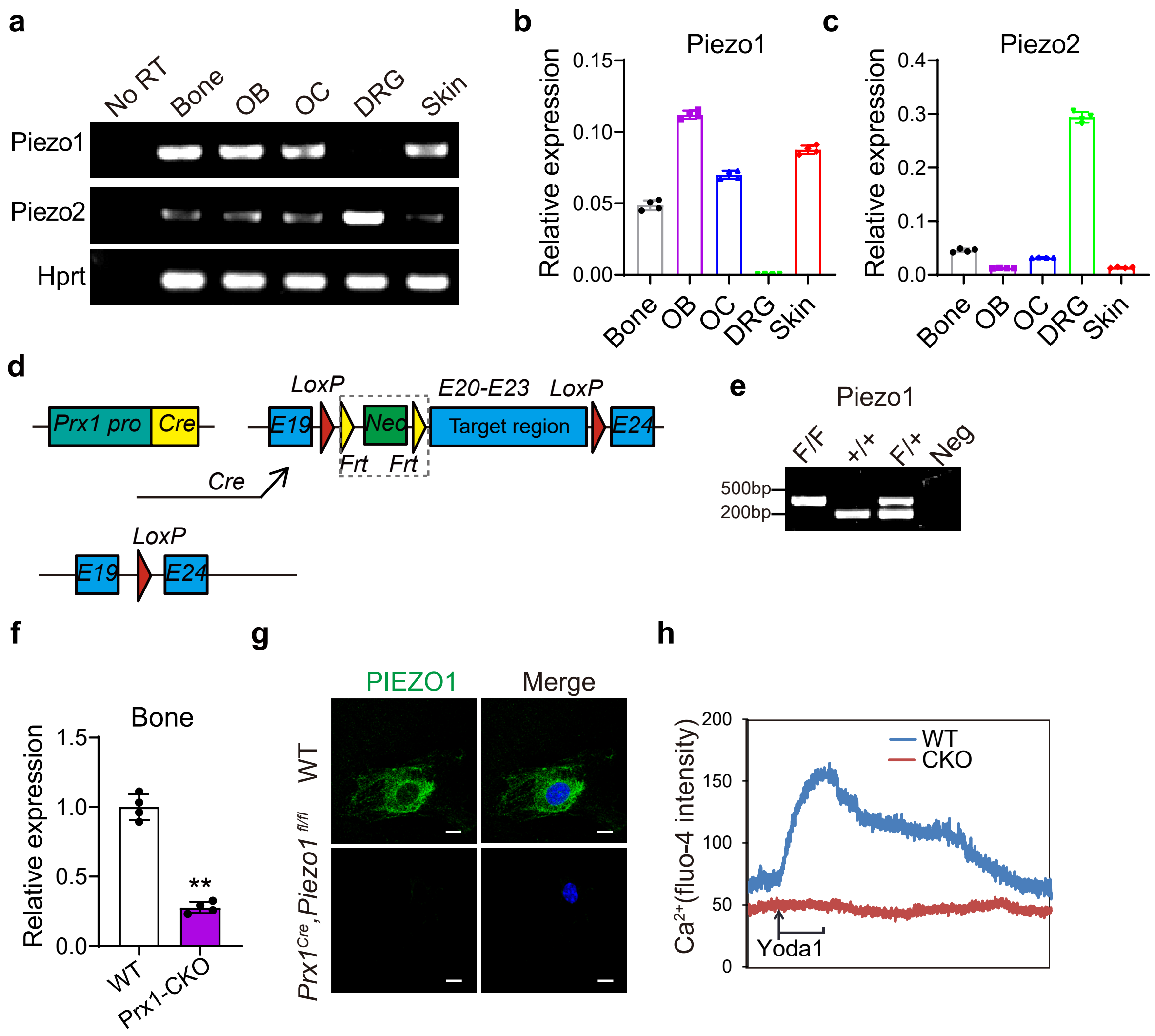

Supplementary Fig. 1 *Piezo1*-deficient bone cells exhibited impaired calcium influx.

a RT-PCR analysis of *Piezo1*, *Piezo2* and *Hprt* transcript levels in the whole bone, osteoblasts (OB), osteoclasts (OC), dorsal root ganglion (DRG) and skin of the mice. **b-c** mRNA expression profiles of *Piezo1* (**b**) and *Piezo2* (**c**) determined by qPCR from the indicated adult mouse tissues. **d** *$Prx1^{Cre}$, $Piezo1^{fl/fl}$* mice construction strategy. **e** Genotyping of the *$Piezo1^{fl/fl}$*, WT and *$Piezo1^{fl/+}$* mice. **f** QPCR analysis of the *Piezo1* expression in the bones of WT and *$Prx1^{Cre}$, $Piezo1^{fl/fl}$* mice. **g** Immunofluorescence assay of PIEZO1 in the osteoblast progenitors isolated from WT and *$Prx1^{Cre}$, $Piezo1^{fl/fl}$* mice. Scale bar = 10 μm. **h** Calcium influx of BMSCs-derived osteoblasts stimulated by Yoda1 (10 μm), cells were isolated from WT and *$Prx1^{Cre}$, $Piezo1^{fl/fl}$* mice. Average trace of all cells (>8 cells) indicated Fluo-4 fluorescence changed over time. Source data are provided in the Source Data File.

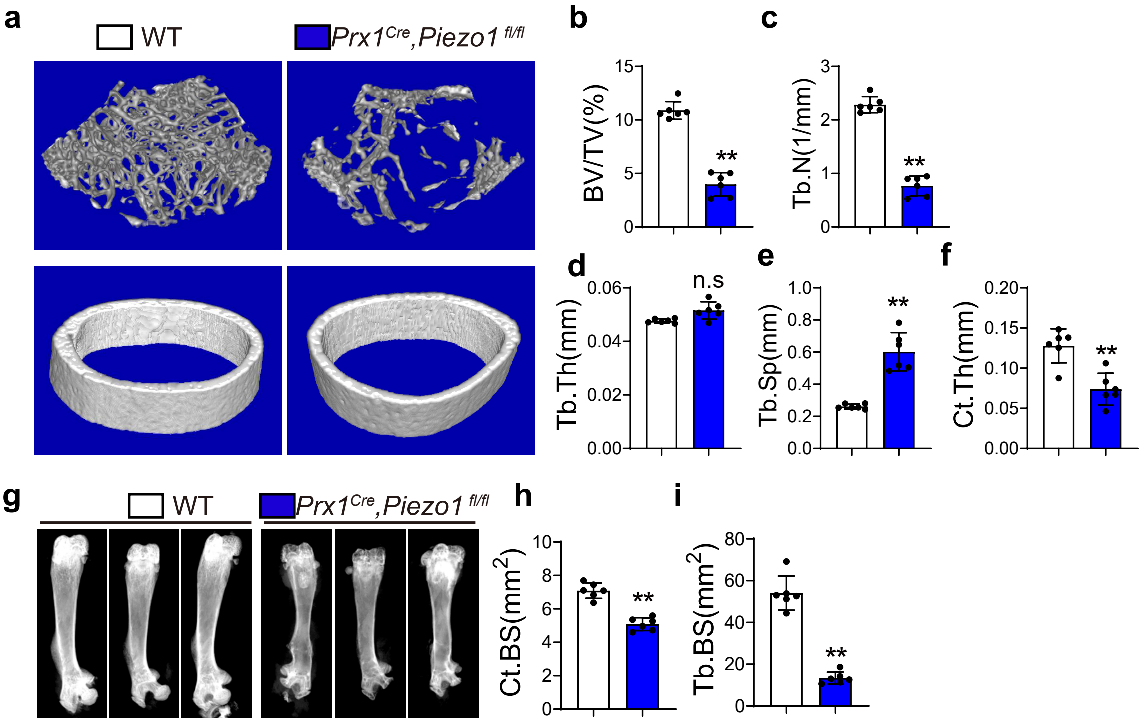

Supplementary Fig. 2 Loss of *Piezo1* in skeletal cells resulted in osteoporosis.
a 3D μ-CT images of trabecular bones of distal femurs isolated from 6-week-old female WT and *Prx1^{Cre}, Piezo1$^{fl/fl}$* mice. **b-f** μ-CT analysis of distal femurs from 6-week-old female WT and *Prx1^{Cre}, Piezo1$^{fl/fl}$* mice for bone volume per tissue volume (BV/TV) (**b**), trabecular number (Tb.N) (**c**), trabecular thickness (Tb.Th) (**d**), trabecular spacing (Tb.Sp) (**e**) and cortical thickness (Ct.Th) of middle shaft of femurs (**f**). Data are mean ± SD, n = 6. **g** X-ray images of the femurs from 6-week-old male WT and *Prx1^{Cre}, Piezo1$^{fl/fl}$* mice. Representative images for 6 independent samples. **h-i** μ-CT analysis of the distal femurs from 6-week-old male WT and *Prx1^{Cre}, Piezo1$^{fl/fl}$* mice for cortical bone surface (Ct.BS) (**h**) and trabecular bone surface (Tb.BS) (**i**). Data are mean ± SD, n = 6. *P < 0.05; **P < 0.01. Two-tailed Student's t-test. Source data are provided in the Source Data File.

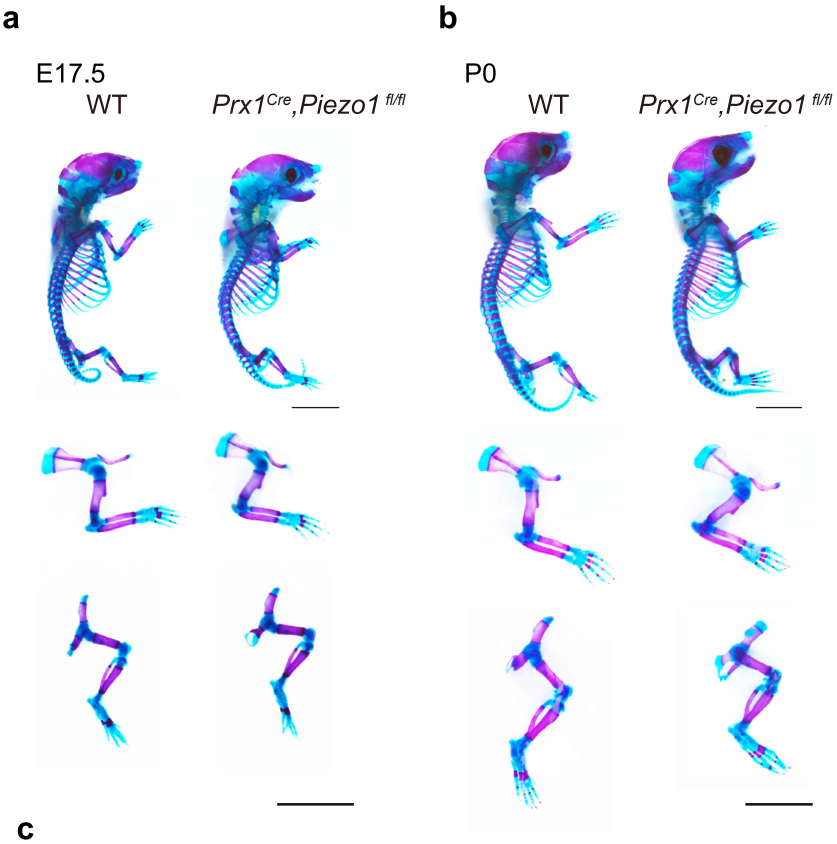

c

Frequency of bone fractures		
	WT(%)	*Prx1* ^{Cre}, *Piezo1* $^{fl/fl}$ (%)
Humerus and ulna	0	88.89
Radius	0	44.44
Scapula	0	11.11
Femur	0	100
Tibia	0	88.89
Rib	0	11.11
Phalanx	0	11.11
	n=8	n=9

Supplementary Fig. 3 Loss of *Piezo1* in bone showed defects after weight-bearing.
a-b Whole mount skeleton staining of WT and *Prx1* ^{Cre}, *Piezo1* $^{fl/fl}$ mice by Alcian blue and Alizarin red S at embryonic day 17.5 (**a**) and postnatal day 0 (**b**). Representative images for more than 3 independent samples Scale bar = 5mm. **c** Quantification of the frequency of fractures occurred in 3-day-old WT and *Prx1* ^{Cre}, *Piezo1* $^{fl/fl}$ mice. WT, n = 8; CKO, n = 9.

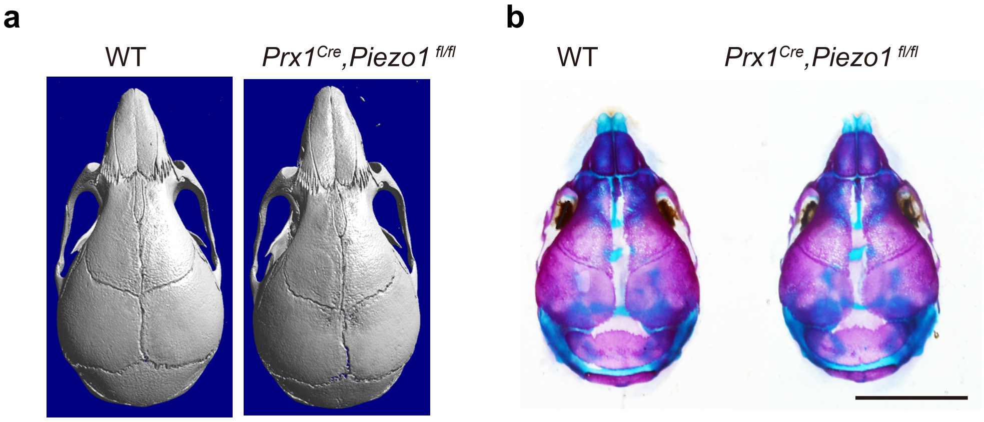

Supplementary Fig. 4 Loss of *Piezo1* did not affect the skull significantly.
a 3D μ-CT images of the cranial bones isolated from 3-week-old male WT and *Prx1*^{Cre}, *Piezo1*$^{fl/fl}$ mice. Representative images for 3 independent samples. **b** Alcian blue and Alizarin red S staining of the skulls of WT and *Prx1*^{Cre}, *Piezo1*$^{fl/fl}$ mice at postnatal day 3. Representative images for more than 3 independent samples. Scale bar = 5mm.

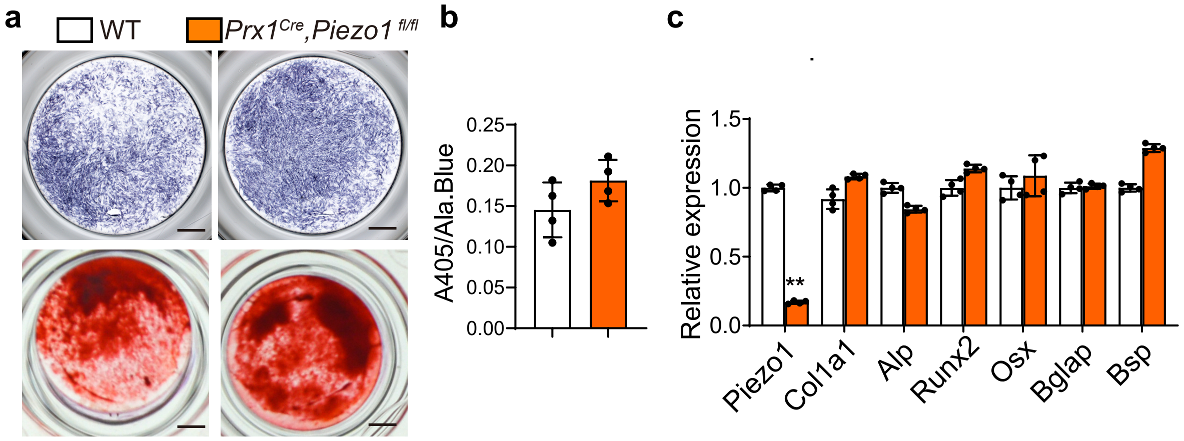

Supplementary Fig. 5 Loss of *Piezo1* exhibited no obvious effect on osteogenesis.
a ALP staining (top) after osteoblast differentiation for 7 days and Alizarin red S staining (bottom) for 21 days. Scale bar = 1mm. **b** ALP activity was measured by phosphatase substrate assay. **c** QPCR analysis of *Piezo1*, *Col1α1*, *Alp*, *Runx2*, *Osx*, *Bglap* and *Bsp* expression after osteoblast differentiation for 7 days, cells were isolated from WT and *Prx1*^{Cre}, *Piezo1*$^{fl/fl}$ mice. *P < 0.05; **P < 0.01. Two-tailed Student's t-test. Data are mean ± SD, n = 4. Source data are provided in the Source Data File.

Supplementary Figure 6

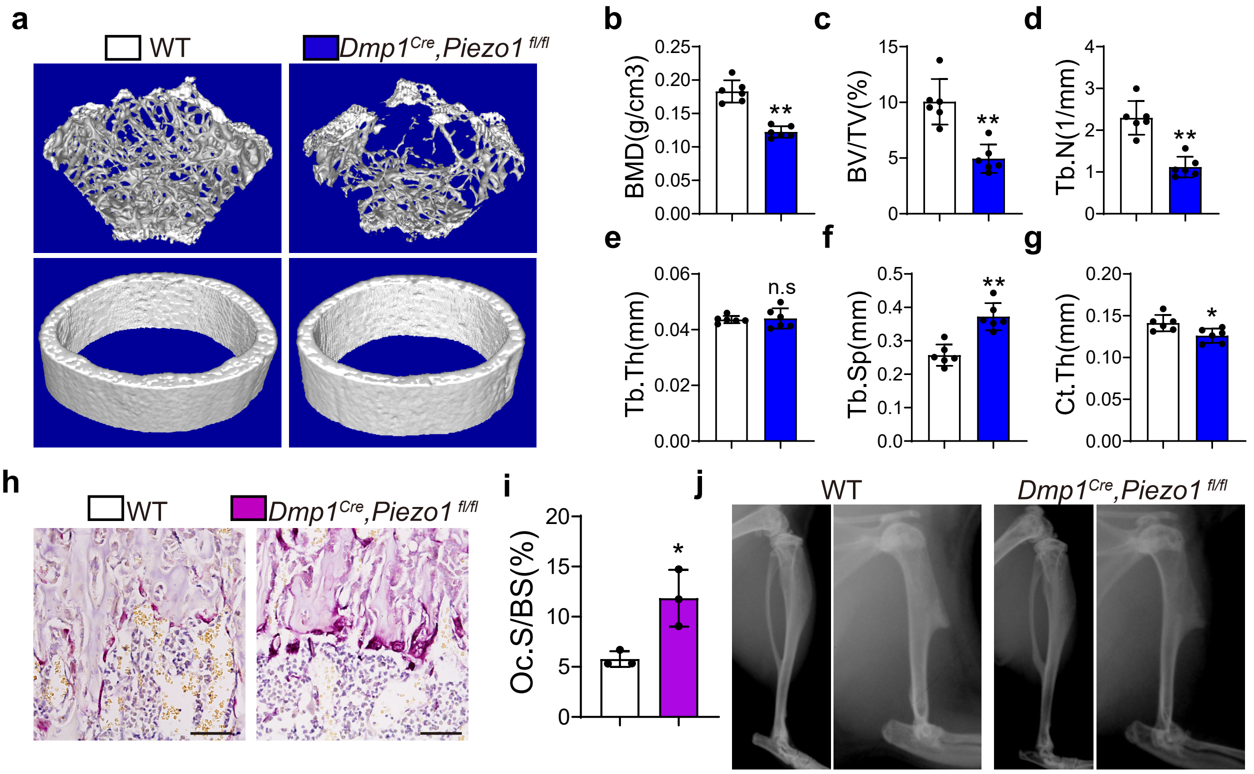

Supplementary Fig. 6 Loss of *Piezo1* in osteocytes affected the bone resorption and bone mass.
a 3D μ-CT images of trabecular bones of distal femurs isolated from 6-week-old female WT and *Dmp1^{Cre}, Piezo1$^{fl/fl}$* mice. (**b-g**) μ-CT analysis of distal femurs from 6-weeks-old female WT and *Dmp1^{Cre}, Piezo1$^{fl/fl}$* mice for bone mineral density (BMD) (**b**), bone volume per tissue volume (BV/TV) (**c**), trabecular number (Tb.N) (**d**), trabecular thickness (Tb.Th) (**e**), trabecular spacing (Tb.Sp) (**f**) and cortical thickness (Ct.Th) of middle shaft of femurs (**g**). Data are mean ± SD, n = 6. **h** TRAP staining of the femurs isolated from 6-week-old WT and *Dmp1^{Cre}, Piezo1$^{fl/fl}$* mice. Scale bar = 50 μm. **i** Quantification of the TRAP staining by osteoclast surface per bone surface (Oc.S/BS). Data are mean ± SD, n = 3. **j** X-ray images of the long bones from 6-week-old male WT and *Dmp1^{Cre}, Piezo1$^{fl/fl}$* mice. Representative images for 3 independent samples. *P < 0.05; **P < 0.01. Two-tailed Student's t-test. Source data are provided in the Source Data File.

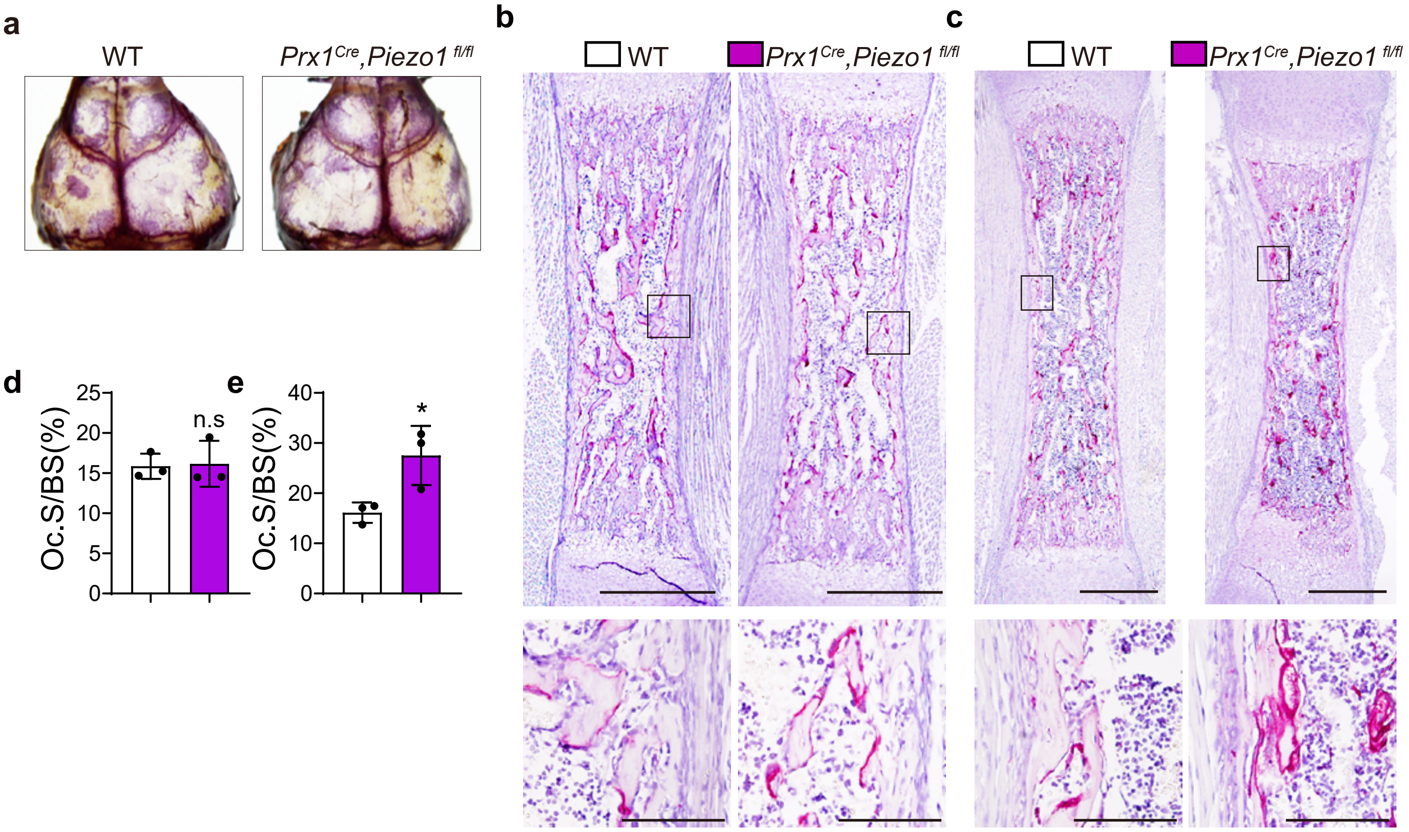

Supplementary Fig. 7 Increased bone resorption caused by loss of *Piezo1* was onset since weight-bearing. **a** Whole-mount TRAP staining for the skulls of 3-week-old male mice. Representative images for 3 independent samples. **b-c** TRAP staining of the femurs isolated from WT and *Prx1*^{Cre}, *Piezo1*$^{fl/fl}$ mice at postnatal day 0 (**b**) and postnatal day 3 (**c**). Scale bars, top, 500 μm; bottom, 100 μm. **d-e** Quantification of the osteoclast surface per bone surface at postnatal day 0 (**d**) and postnatal day 3 (**e**). *P < 0.05; **P < 0.01. Two-tailed Student's t-test. Data are mean ± SD, n = 3. Source data are provided in the Source Data File.

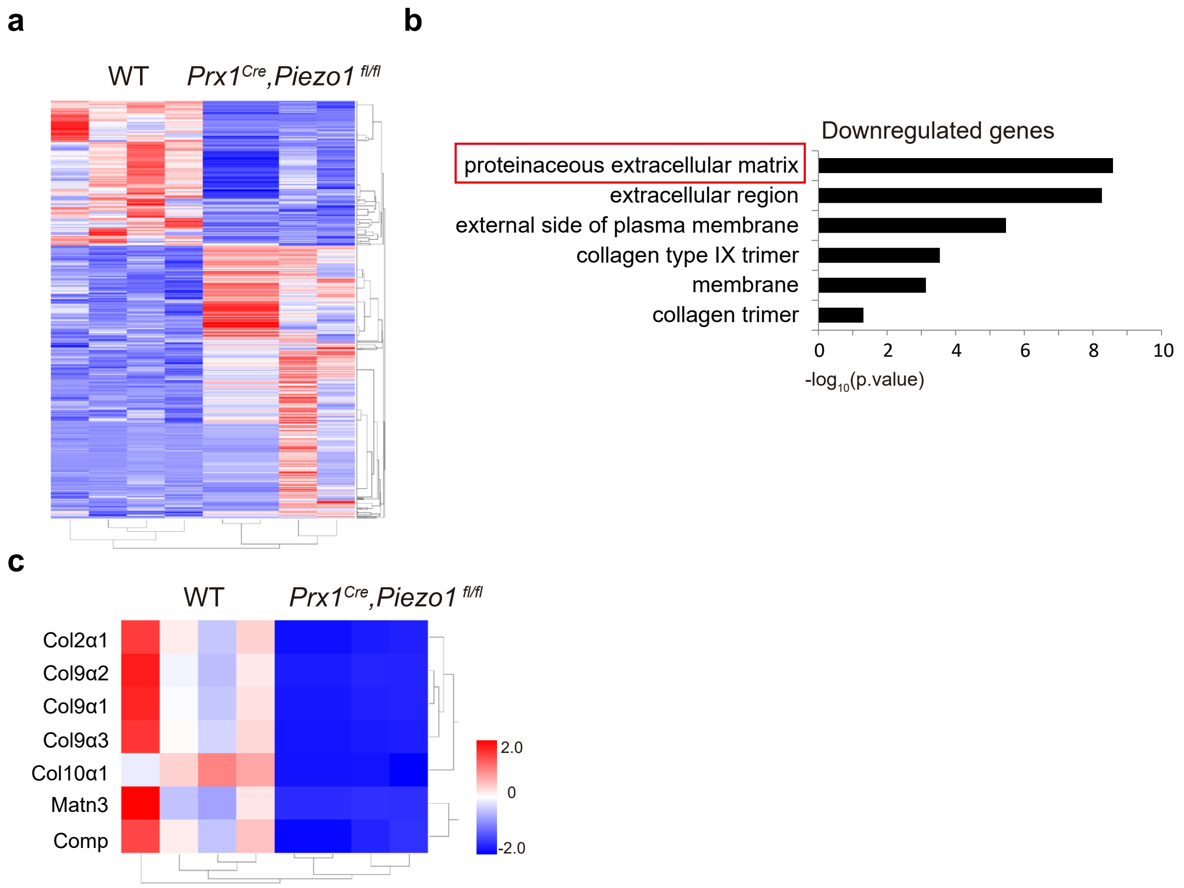

Supplementary Fig. 8 Extracellular proteins were dysregulated in *Prx1*^{Cre}, *Piezo1*$^{fl/fl}$ mice.
a Heat map of the genes (FoldChange > 1.5, P.value < 0.05) from RNA-seq data (GSE135282) of the cortical bones isolated from the 3-week-old WT and *Prx1*^{Cre}, *Piezo1*$^{fl/fl}$ mice to compare the difference of gene expression. The genes were ordered by clustering tightness. Each row represented a single RNA-seq data, n = 4. **b** Gene ontology (GO) enrichment analysis of downregulated genes (FoldChange > 1.5, p.value < 0.05). **c** Heat map of extracellular matrix genes from RNA-seq data of cortical bone isolated from 3-week-old WT and *Prx1*^{Cre}, *Piezo1*$^{fl/fl}$ mice. The genes were ordered by clustering tightness. Each row represented a single RNA-seq data, n = 4.

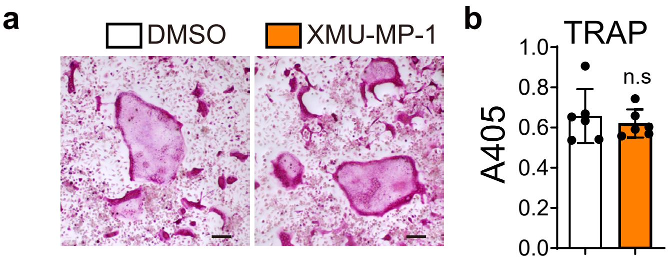

Supplementary Fig. 9 Xmump1 did not inhibit osteoclast differentiation.

a Bone marrow monocytes were seeded in 96-well plates and treated with 20 ng/ml M-CSF and 250 ng/ml RANKL for 6 days with DMSO or XMU-MP-1 (0.2 μm). **b** Quantification of the osteoclast differentiation by TRAP activity of the culture supernatants. *P < 0.05; **P < 0.01. Two-tailed Student's t-test. Data are mean ± SD, n = 6. Source data are provided in the Source Data File.

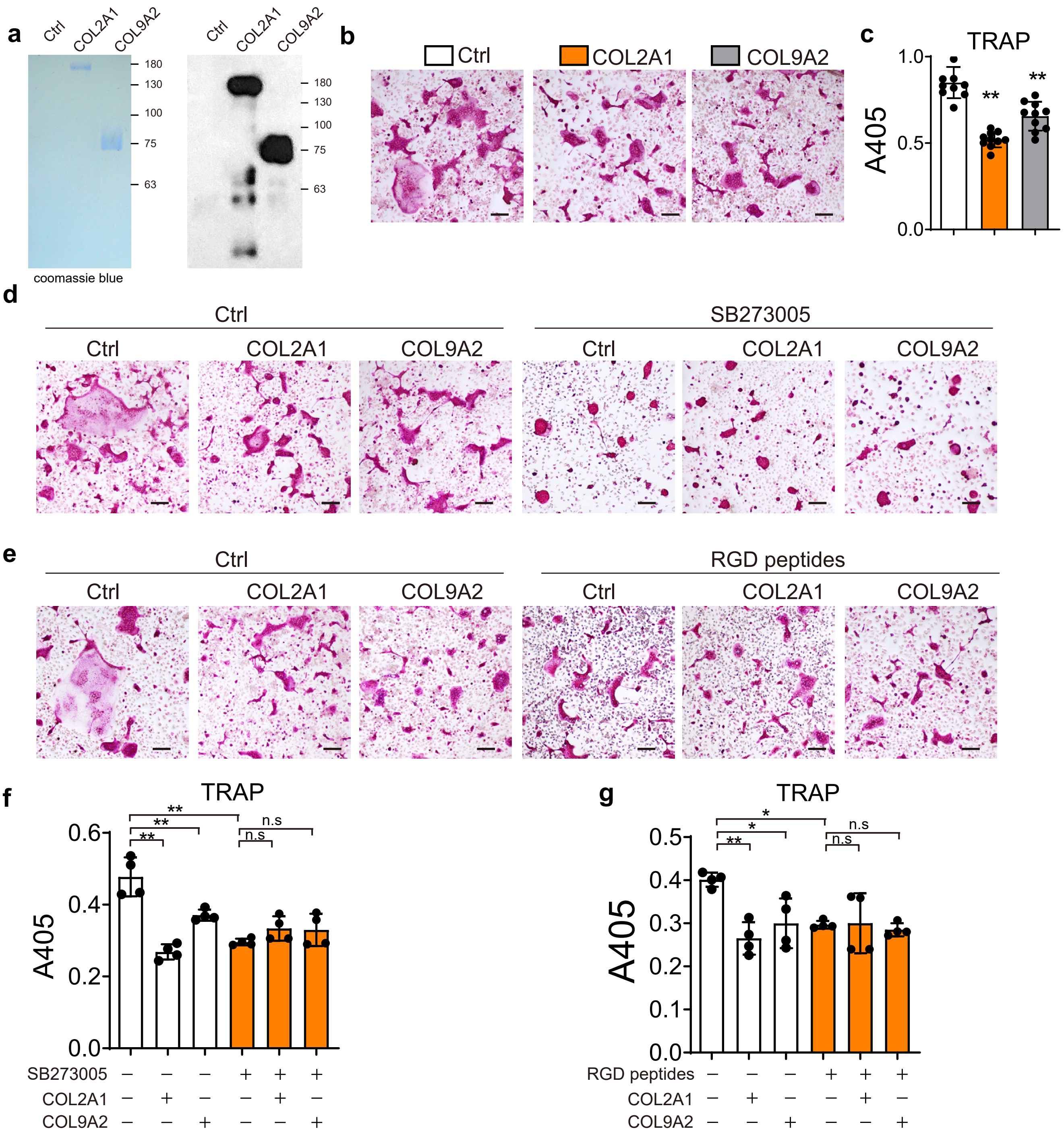

Supplementary Fig. 10 COL2α1 and COL9α2 inhibited osteoclastogenesis through integrin pathway. **a** Analysis of COL2α1 and COL9α2 enriched from the supernatants of 293T cells transfected with control, *Col2α1* or *Col9α2* plasmids by coomassie blue staining (left) or western blot of FLAG antibody (right). **b** Bone marrow monocytes were seeded in 96-well plates and treated with 20 ng/ml M-CSF and 250 ng/ml RANKL for 6 days with indicated collagens or control. Scale bar = 100 μm. **c** Quantification of the osteoclast differentiation by TRAP activity of the culture supernatants from (**b**). Data are mean ± SD, n = 10. **d-e** Bone marrow monocytes were seeded in 96-well plates and treated with 20 ng/ml M-CSF and 250 ng/ml RANKL for 6 days with indicated treatment. Scale bar = 100 μm. **f-g** Quantification of the osteoclast differentiation by TRAP activity of the culture supernatants from (**d-e**). Data are mean ± SD, n = 4. *P < 0.05; **P < 0.01. Ordinary one-way ANOVA. Source data are provided in the Source Data File.

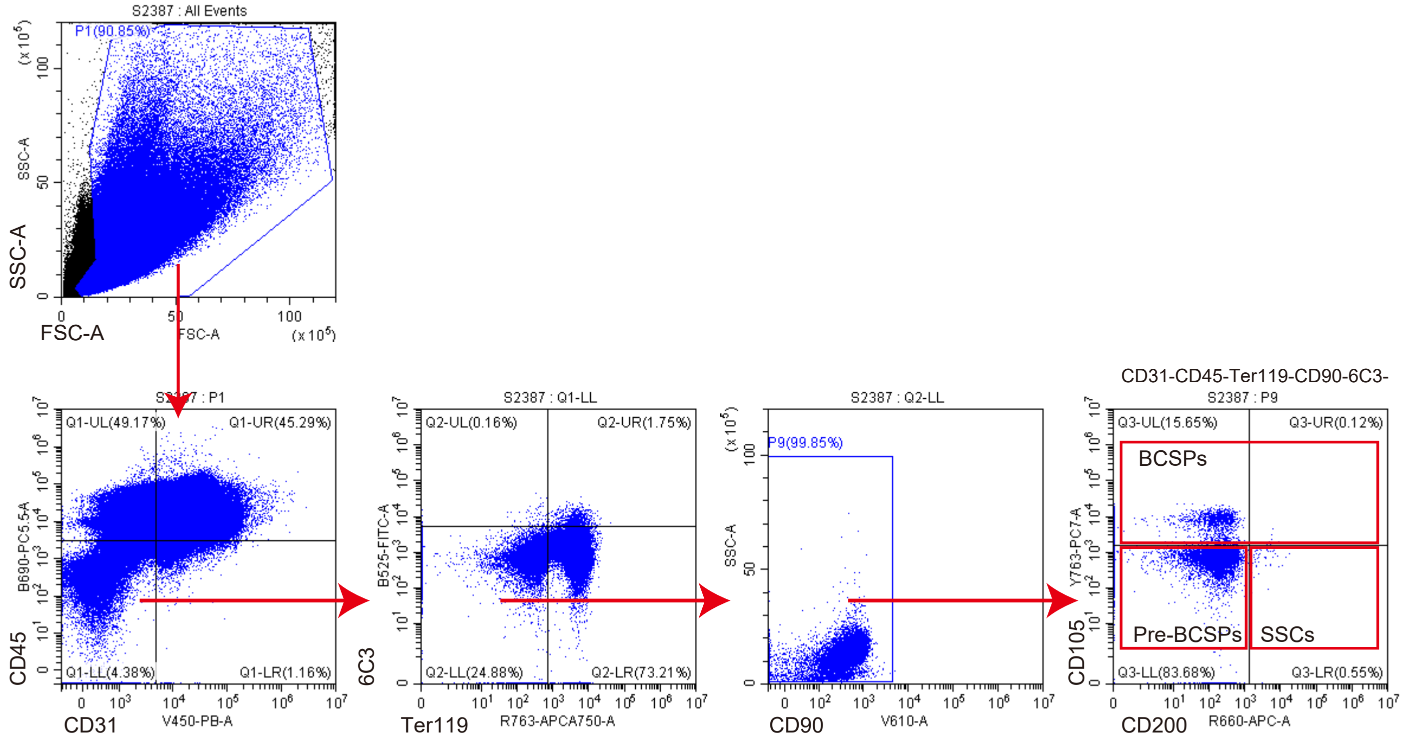

Supplementary Fig. 11 Gating strategy.

Live cells were gated on SSC-A/FSC-A, and then SSCs (CD105- CD200+), BSCPs (CD105+) and pre-BSCPs (CD105- CD200-) were gated from CD31-CD45-Ter119-CD90-6C3-live cells. The boundary between "positive" and "negative" was defined by the single positive control and the negative control.
